# The Various Roles of Fatty Acids

**DOI:** 10.3390/molecules23102583

**Published:** 2018-10-09

**Authors:** Carla C. C. R. de Carvalho, Maria José Caramujo

**Affiliations:** 1 Institute for Bioengineering and Biosciences, Department of Bioengineering, Instituto Superior Técnico, Universidade de Lisboa, Av. Rovisco Pais, 1049-001 Lisboa, Portugal; 2 Centre for Ecology, Evolution and Environmental Changes, Faculdade de Ciências, Universidade de Lisboa, Campo Grande, Edifício C2-5º Piso, 1749-016 Lisboa, Portugal; mj.caramujo@fc.ul.pt

**Keywords:** fatty acid synthesis, cellular membranes, membrane remodelling, biomarkers, omega-3 fatty acids, specialized lipids, glycerophospholipids, storage lipids, lipid bodies, lipidomics

## Abstract

Lipids comprise a large group of chemically heterogeneous compounds. The majority have fatty acids (FA) as part of their structure, making these compounds suitable tools to examine processes raging from cellular to macroscopic levels of organization. Among the multiple roles of FA, they have structural functions as constituents of phospholipids which are the “building blocks” of cell membranes; as part of neutral lipids FA serve as storage materials in cells; and FA derivatives are involved in cell signalling. Studies on FA and their metabolism are important in numerous research fields, including biology, bacteriology, ecology, human nutrition and health. Specific FA and their ratios in cellular membranes may be used as biomarkers to enable the identification of organisms, to study adaptation of bacterial cells to toxic compounds and environmental conditions and to disclose food web connections. In this review, we discuss the various roles of FA in prokaryotes and eukaryotes and highlight the application of FA analysis to elucidate ecological mechanisms. We briefly describe FA synthesis; analyse the role of FA as modulators of cell membrane properties and FA ability to store and supply energy to cells; and inspect the role of polyunsaturated FA (PUFA) and the suitability of using FA as biomarkers of organisms.

## 1. Fatty Acid Synthesis

Fatty acids (FA), as part of molecules or acting individually, have diverse functions in cells that range from structural “building blocks” of cell membranes to suppliers of energy and signalling molecules (Table 1). The FA in cells derive either from exogenous sources or from *de novo* FA synthesis. Some organisms require some physiologically essential FA compounds that either cannot be synthesized *de novo*, or cannot be synthesized in sufficient quantities to meet the organism demands for general metabolic functioning, somatic growth and reproduction. 

The pathway for FA biosynthesis is highly conserved within the kingdoms of life, starting with the formation of malonyl-CoA by carboxylation of acetyl-CoA and further condensation of malonyl-CoA with acetyl-CoA with the release of CO_2_ [1]. Different enzymes and different genetic organizations have nevertheless evolved to reach the similarities in the general pathway. In animals and fungi, all steps of FA biosynthesis involve one multifunctional protein complex, the type-I fatty acid synthase (FAS), which in divided into the fungal type-Ia FAS and the type-Ib FAS in animals [2,3]. In most prokaryotes and in the plastids of plants (where *de novo* synthesis of plant FA occurs), FAS is of the type-II [4,5,6]. The exception for prokaryotes pertains to the Gram-positive, mycolic acid-producing bacteria, which contain a type-I FAS with the ability of start *de novo* FA biosynthesis and an additional type-II FAS, which is only involved in the elongation of FA with medium chain length [7,8,9]. Studies on *de novo* synthesis of FA in Archae are rare. Archaeal membrane phospholipids are considered to incorporate isoprenoids instead of FA [10,11] although experimental data has shown that FA do exist in archaea [12] and a type-II FAS may be involved in their synthesis [13,14,15].

Photosynthetic marine and freshwater microalgae, heterotrophic protists and bacteria have been considered to be the producers of natural ω3 long-chain (≥20 carbons) polyunsaturated fatty acids (PUFA), because they possess the key enzymes including methyl-end (or “ωx”) desaturases necessary for their *de novo* synthesis [16,17,18,19,20,21]. Plants are recognised as having limited elongation capacity to produce long-chain PUFA, having the ability for *de novo* synthesis of PUFA of up to 18 carbons [17]. It is considered that all PUFA in food webs thus originate from primary producers and animals have only the ability to modify them by bioconversion and elongation as they pass through the food web (i.e., trophic upgrading). Recent studies have identified 121 ωx desaturase sequences from 80 species within various invertebrate taxa suggesting that, in addition to trophic upgrading, *de novo* synthesis of PUFA is possible in some invertebrates [22,23]. These studies point to the need of a major revision in the scientific understanding of ω3 long-chain PUFA production in global food webs. Nevertheless, for vertebrates, PUFA are essential dietary nutrients with, for example, fish having the ability to contribute to trophic upgrading by metabolizing C18 PUFA, 18:3ω3 (α-linolenic acid, ALA) and 18:2ω6 (linoleic acid, LA) to long chain PUFA, with the ability to produce 20:5ω3 (eicosapentaenoic acid, EPA) or 22:6ω3 (docosahexaenoic acid, DHA) dependent upon species [22,24,25,26,27]. Freshwater fish are reported to have a greater ability for conversion of C18 PUFA to long chain PUFA than marine species, which is attributed to deficiencies in at least one key enzyme of the long chain PUFA biosynthesis pathway in marine fish [28,29].

In humans, although some controversy remains as to the extent ALA assures the maintenance of tissue long chain ω3 PUFA in human infants, including DHA [30], there is mounting evidence that “indeed dietary ALA is a crucial dietary source of ω3 fatty acids and its dietary inclusion is critical for maintaining tissue long chain ω3 levels” [31]. DHA can originate directly from the diet or, in the liver, ALA is converted to DHA and exported to the blood bound to serum albumin, either as an unesterified FA or esterified as DHA-lysophosphatidylcholine [32,33,34]. Both ω6 and ω3 PUFA are essential for human nutrition and a balance between the ingestion of ω6 to ω3 FA is fundamental for maintaining health. Unbalanced diet favouring ω6 PUFA has prothrombotic and proinflammatory implications, which contributes to the prevalence of atherosclerosis, obesity and diabetes in humans [35,36,37,38,39]. Acknowledgement of the importance of adequate intake of FA in humans has been translated in the setting of dietary reference values for lipids by the European Union: the intake of *trans* and saturated FA should be kept as low as possible; the adequate intake (AI) for LA and ALA is set at 4% and 0.5%, respectively, of total energy intake; and the AI is set between 100–250 mg for EPA + DHA according to age (i.e., infants, children and adults) and reproductive stage of women [40].

## 2. Fatty Acids as Modulators of Membrane Properties

### 2.1. In Prokaryotes

In prokaryotes, the cellular envelop has the fundamental role of protecting these organisms from the surrounding environment. Gram-negative bacteria have a thin peptidoglycan cell wall which is surrounded by an outer membrane rich in lipopolysaccharides whilst Gram-positive bacteria do not have an outer membrane but possess a thick peptidoglycan layer in their cell walls. The phylum actinobacteria, which include genera such as *Rhodococcus*, *Mycobacterium* and *Nocardia*, have cell walls containing mycolic acids which are complex hydroxylated branched-chain long FA. Protecting and surrounding the cytoplasm in all cells is a phospholipid bilayer that forms the cellular membrane. The amphiphilic character of cytoplasmatic membranes derives from the phospholipids which have a polar head group and two hydrophobic FA tails. These molecules form spontaneously bilayers in aqueous environments [41].

In Archaea, the lipids contain distinctive condensed isoprenyl units connected to the *sn*-glycerol-1-phosphate backbone by ether bonds [11,42]. In prokaryotic and eukaryotic cells, the carbon chains are mainly linear but archaeal lipids are branched every fourth carbon, with a single methyl group linked to these carbon atoms. The unique structure of archaeal lipids and their stereo specificity was hypothesized to be responsible for the ability of these organisms to resist and thrive under extreme environmental conditions [43]. Nevertheless, archaeal strains have also been found in non-extreme environments and bacteria in extreme ones. In fact, bacteria may produce membrane ether lipids [44] and archaea may also produce non-ether-linked FA [12]. This indicates that prokaryotes modulate their membrane according to the environmental conditions.

The ability of microorganisms to maintain their biological functions under stressful environmental conditions may involve changes in their protein, sterol, hopanoid and carotenoid content, yet it is mainly accomplished through changes produced in the lipid composition of their cellular membranes [45,46,47,48]. Since one of the mechanisms that cells may use to generate metabolic energy occurs at the membrane where energy transducing systems convert chemical (or light in phototrophs) energy into electrochemical energy or vice-versa [48], the integrity of cellular membranes is paramount for cell survival. By changing the FA composition of the membrane phospholipids, the cells try to maintain membrane fluidity through a mechanism called “homeoviscous adaptation” [26,27]. This may be achieved by *de novo* synthesis of membrane lipids or acyl chain remodelling of the FA of the existing phospholipids of the cellular membrane [27].

Each bacterial strain possesses a unique fatty acid profile, when grown on a given culture medium at a certain temperature, allowing their identification as explained in Section 5.1. However, when the growth conditions change, prokaryotic cells can alter their FA composition to adjust the fluidity of the membrane by a series of strategies (Table 2).

In response to e.g., temperature changes, the cells try to maintain the fluidity of the cellular membrane by changing the FA composition which will affect the transition temperature that in turn mark an order-disorder transition of lipid bilayers [70,71,72]. At phase-transition temperature, 50% of the hydrocarbon chains melt and both a liquid crystalline phase and a rigid gel phase may be observed. In the gel phase, the phospholipids hydrocarbon chains are fully extended and aligned perpendicularly to the plane of the bilayer resulting in a closely ordered side-to-side packing [73]. In the liquid crystalline state, both long-range order and short-range disorder occurs as the bilayer structure is maintained by electrostatic interactions but acyl chains may flap around [73]. In the latter phase, the cross-sectional area per phospholipid molecule increases to ca. 6–7 nm^2^ while the bilayer thickness decreases to ca. 4.0–4.5 nm, increasing the permeability of the membrane and allowing intra- and intermolecular rotational and lateral motion [73,74]. This provides a functional state for many biochemical processes, and permeability to neutral molecules such as water, oxygen and carbon dioxide but restricted access to ions and solutes [72].

Microorganisms adjust the transition temperature of the lipids by making changes in the hydrocarbon length, unsaturation degree, charge and headgroup of phospholipids (Table 2). Shorter and unsaturated fatty acids present lower melting points when compared to their, respectively, longer and saturated counterparts. The uncharged phospholipid phosphatidylethanolamine has higher melting temperature than phosphatidylcholines and also higher than the negatively charged phosphatidylserine, phosphatidylglycerol or phosphatidylinositol with corresponding acyl chain compositions [75,76]. The more charged a membrane is, the lower is its melting temperature [76].

Most of the studies regarding prokaryote adaptation at the cellular level investigate the modifications in phospholipids when cells grow at different temperatures. Adaptation to cold shock has been extensively studied in *Escherichia coli* and *Bacillus subtilis*, where the first membrane phospholipid desaturase induced by cold in a non-photosynthetic organism was reported [77,78,79]. While *E. coli* responds to a decreasing growth temperature by increasing the amount of unsaturated FA (16:1ω7 is also converted to 18:1ω7), *B. subtilis* cells induce the production of membrane phospholipid desaturase leading to an increase in unsaturated FA and also increase *anteiso*-branched FA [80,81,82]. The food-borne pathogen *Listeria monocytogenes*, capable of growing at refrigerating temperatures, contains an unusual FA profile dominated by more than 95% branched-chain FA and relies on an increased *anteiso*-15:0 proportion for adaptation to low temperatures [83]. Branched-chain FA include *iso*-, *anteiso*- and ω-aliphatic FA. In the *iso*-structure the methyl group is located at the penultimate carbon atom while in the *anteiso* form, the methyl group is at the antepenultimate carbon from the end. While a straight saturated FA and an *iso*-FA with the same number of carbons present similar melting points, the phase transition temperature of the iso-acyl is lower: for example, the iso-acyl phosphatidylcholine has a phase transition temperature of 18 to 28 °C below that of the corresponding saturated acyl phosphatidylcholine [84]. *Anteiso*-FA present phase transition temperatures below that shown by their *iso* counterparts: for example, *iso*-15:0 has a transition temperature of 52.2 °C whilst *anteiso*-15:0 presents a value of 25.8 °C [85]. Curiously, odd-numbered *anteiso*-branched phosphatidylcholines present a low-temperature gel phase that is the reflection of a highly ordered condensed phase, similar to that of linear saturated *n*-acyl phosphatidylcholines, which is not observed in even-numbered *anteiso*-branched phosphatidylcholines [86].

The FA composition of *Bacillus* cells is an essential criterion to define their species, with some FA being common at the genus level whereas others are specific to species from certain ecological niches. It has been proposed that it is possible to predict the thermotype of strains of *B. cereus* based on the *iso*-15:0/*iso*-13:0 ratio and proportion of 16:1ω11 and 16:1ω6 [87] while the *anteiso*-15:0/*iso*-15:0 ratio may differentiate between psychrophilic, mesophilic and thermophilic *Bacillus* species [78]. Thermophilic *Bacillus* sp. present higher contents of lower-melting *iso*-branched FA accompanied by lower amounts of lower-melting *anteiso*-branched FA and longer average FA chain length than mesophilic *Bacillus* sp. [85]. *B. megaterium* cells responded to decreasing temperature using a biphasic behaviour: saturated straight-chain and *iso*-branched FA decreased as the temperature decreased from 40 to 20–26 °C and *anteiso*-branched FA content decreased from 20–26 °C to 10 °C, with unsaturated FA increasing as temperature decreased from 40 to 10 °C [88].

Certain bacterial species belonging to the genera *Pseudomonas* and *Vibrio* possess an alternative adaptive mechanism when growth is inhibited: they are able to isomerize unsaturated FA from *cis* to *trans* configuration of the double bond without a shift in its position [89,90,91]. In *Vibrio* sp. ABE-1 cells, the unique FA 16:1ω7*trans* is located at the *sn-*2 position of phosphatidylethanolamine (PE) and the *sn*-1 position may contain the FA 16:1ω7*cis* or 16:0 [91]. The transition temperatures for 16:1ω7*cis*/16:1ω7*trans*-PE and 16:0/16:1ω7*trans*-PE, which were predominantly produced when the cells grew at 5 °C in the presence of a growth inhibitor, are −3 °C and 38 °C, respectively. The transition temperatures are, respectively, 31 and 18 °C higher than those for 16:1ω7*cis*/16:1ω7*cis*-PE and 16:0/16:1ω7*cis*-PE.

The same *cis-trans* isomerization strategy is used by Gram-negative cells as response to toxic compounds such as organic hydrocarbons. In *Pseudomonas*, this short-term response results in a denser membrane packing and gives time to the cells for *de novo* biosynthesis of membrane components necessary for a better and broader adaptation [92]. Synthesis of *trans* FA completes within 30 min following exposure to the stress by direct isomerization of the *cis* isomer, without changes in the position of the double bond [89,90]. In *P. putida* Idaho exposed to xylene, *trans*-unsaturated FA were observed after 5 min and maximum amount was observed after 30 min whilst the increase in saturated FA was observed 15 min after exposure to xylene, with maximum content being reached after 2 h [93].

Besides the fast isomerization of FA in Gram-negative bacteria, another fast and effective response to stress, such as heat shock, pH changes or the presence of organic solvents, is the release of outer membrane vesicles (OMVs) from the cell surface [61,62,94]. OMVs in *P. aeruginosa* are released following exposure to antibiotics and are involved in the release of virulence factors during infection of human cells [95]. *P. putida* DOT-T1E responded to the presence of toxic concentrations of long-chain alcohols, NaCl induced osmotic stress, heat shock and the presence of EDTA by releasing OMVs within 10 min following exposure to stress resulting in a highly hydrophobic cell surface [96]. The OMVs contained mainly 16:0 and 18:1ω7*cis* and also a considerable amount of 18:0.

Until the 1990s, it was considered that bacteria were unable to produce PUFA, with the exception of certain cyanobacteria but it is now accepted that long-chain PUFA are produced by marine bacteria [60,97,98]. In fact, PUFA are quite important for the maintenance of membrane fluidity in prokaryotes living in deep-sea, under high pressure and low temperature [52,97]. The production of eicosapentaenoic acid (EPA, C20:5ω3) was demonstrated in marine bacteria following isolation of ca. 50,000 bacterial strains from the intestines of several marine fish and animals [49]. One of the strains, SCRC-2738, which was isolated from a horse mackerel and found to be phylogenetically similar to *Shewanella putrefaciens*, was able to produce 25–40% of total FA as EPA. The strain was later identified as *S. pneumatophori* SCRC-2738 and an “EPA biosynthesis gene cluster” containing five genes *pfaA*, *pfaB*, *pfaC*, *pfaD* and *pfaE*, was identified as necessary for EPA synthesis [50]. In two barophilic bacteria, DB21MT-2 and DB21MT-5, the phospholipids detected phosphatidylglycerol, phosphatidylinositol, phosphatidylcholine, diphosphatidylglycerol and phosphatidylethanolamine and its methylated forms phosphatidylmethylethanolamine and phosphatidyldimethylethanolamine, contained PUFA with five or six double bonds mainly attached on the *sn*-2 position [51]. Besides, it was observed that the PUFA were associated with almost every phosphatidylglycerol molecule in these extremely barophilic bacteria from the Marianas Trench at 11,000 m. The lower phase transition temperature of phosphatidylglycerol (20–30 °C lower than that of phosphatidylethanolamine) and the high concentrations of PUFA allow the adaptation of barophilic bacteria to the low temperature and high hydrostatic pressure of the deep-sea environment [51,99]. Although, a relation between the ability to produce PUFA and psychrophilic and/or piezophilic growth strongly suggest a role of PUFA in bacterial membrane adaptation, a mutant of *Photobacterium profundum* unable to produce PUFA could grow under high pressure and low temperature conditions [100]. In this case, the EPA-deficient strain increased the content of monounsaturated FA 16:1 and 18:1 which could compensate the lack of EPA to reach the desired membrane fluidity.

PUFA have been mainly reported in marine bacteria, but recently, the existence of PUFA has also been reported in bacteria isolated from soil samples or from shallow aquatic samples. The Gram-positive *R. erthropolis* DSM 1069 responded to osmotic stress caused by NaCl within the 35 min after the addition of salt [59]. Modifications in the FA profile could be observed after only 6 min: the amount of monounsaturated FA decreased to ca. half of the amount observed in unchallenged cells and they started to produce hydroxy-substituted, saturated methyl-branched, saturated cyclopropyl-branched and PUFA at concentrations ranging between 7.8 and 14.7% of the total FA. The production of PUFA was surprising and it reached more than 36% of total FA when the cells were exposed for 35 min to concentrations higher than 5.5% NaCl. Since the appearance of PUFA was accompanied by a decrease in the percentage of monounsaturated FA, constitutively expressed FA desaturases should be responsible for the synthesis of PUFA. Strain *R. erythropolis* DCL14 produced PUFA when the cells were adapted to conditions previously considered as extreme for non-adapted cells: when the cells grew at 4 °C, at pH 4–10 (but not at pH 3 or 11) and in the presence of challenging amounts of NaCl and CuSO_4_ in the growth medium [60]. It was recently reported that *R. aetherivorans* BCP1 produces PUFA when using naphthenic acids as sole carbon and energy sources [101].

Other changes observed in FA composition during environmental adaptation involved e.g., the synthesis of branched-FA, cyclopropane FA or other specialized lipids. Methyl branched FA present a biphasic dependence of chain-melting temperature on the position of the methyl substitution because this single group will divide the lipid chain into a longer and shorter section [102]. Methyl branching reduces lipid condensation, decreases lipid bilayer thickness, lowers chain ordering and enhanced the fluidity of the membrane through the formation of kinks at the branching point [103]. Cyclopropane FA reduce the fluidity of the cellular membrane by modulating lipid packing, enhancing the formation of gauche defects and increasing lipid diffusion [103]. Nevertheless, cyclopropane FA are in general more ordered than the corresponding unsaturated chains which could explain how these FA may enhance the stability of the membrane e.g., at high temperatures and simultaneously reduce its permeability to toxic compounds.

Besides being observed in several bacteria when the cells enter the stationary phase, the proportion of cyclopropyl-branched FA is changed as response of e.g., *R. erythropolis* cells to both low and high pH values [60], to acid shock in *E. coli* [104] and resistance of *Salmonella enterica* serovar Typhimurium (*S. typhimurium*) to low pH values [105]. Incorporation of cyclopropane FA from the medium or by introduction of a functional *cfa* gene in *cfa* mutant strains of *E. coli* [104] and *S. typhimurium* [105] restore the resistance of these cells to low pH. Since cyclopropane ring formation in bacterial membranes has high energetic cost and occurs immediately before cellular growth ceases, it could be an indication of the importance of these FA for cell adaptation to the adverse conditions found in the stationary phase, yet scientific evidence is still missing [106].

The studies on the FA composition of the cell membrane of prokaryotes demonstrate the panoply of responses that the cells may use to adjust the fluidity of the cellular membrane when placed under challenging conditions. From enzymatic changes of FA to *de novo* synthesis, the cells try to make the most rapid changes using the least energy possible. Nevertheless, FA in the environment themselves can pause a threat to the stability and function of bacterial cell membranes. Whilst their antibacterial mode of action is poorly understood, free FA acting upon the cell membrane can disrupt the electron transport chain and oxidative phosphorylation [107]. FA as free FA or in monoacylglycerides are promising antibacterial agents with possible therapeutic applications for human health and medicine [108].

### 2.2. In Eukaryotes

The eukaryotic cell membrane forms a selective barrier controlling the transport of molecules into and from the cell, regulates cell communication possesses and is involved in numerous complex functions, including proliferation, differentiation, secretion, migration, invasion and phagocytosis. In eukaryotes, in addition to protecting the cell from the surrounding environment, membranes are present at the subcellular scale, forming, for example, the endoplasmic reticulum, surrounding the cell nucleus and various types of organelles. This contributes to the great compositional diversity of membranes in eukaryotes, further expanded in multi-cellular organisms where specific compositions of the membranes are required to fulfil specific tissue functions (see [109]). This section focuses on the role of fatty acids regulating the properties of plasma membranes under various environmental conditions. FA composition modulate membrane biophysical properties of biological membranes as part of membrane lipids that is, phospholipids, phosphosphingolipid, sphingolipids and gangliosides. These lipids, together with cholesterol, compartmentalize and coalesce in cell membranes, organelle membranes, bilayer leaflets forming a mosaic of gel and fluid domains coexisting in the plane of the lipid bilayer [110,111]. The chain length and degree of unsaturation, double bond position and hydroxylation of FA integrated in phospholipids and sphingolipids determines their impact on the membrane biophysical properties: saturated fatty acids with short chains form less viscous membranes while mono-unsaturated fatty acids and ω6 and ω3 PUFA form more fluid membranes than saturated FA [112]. As in prokaryotes, the control of membrane fluidity is especially important in poikilotherms eukaryotes facing temperature stress.

The response of poikilotherms to alterations in environmental temperature is not uniform (see [113]). Adjustments of membrane fluidity to the different growth temperatures often imply alterations in sterol and glycerolipid class relative proportions (i.e., alterations in headgroup composition of phospholipids) in membranes [114]. The shortening of hydrocarbon chain length or the introduction of a *trans*- or *cis*-double bond into a saturated hydrocarbon chain are, however, widespread mechanisms employed by poikilotherms to maintain lipid order at physiologically advantageous values. Experiments with yeast for instance, have shown that some species (e.g., *Saccharomyces cerevisiae*) increase FA chain length with increasing temperature while others (e.g., *S. toruloides*) decrease the degree of FA unsaturation and others (*C. utilis* and *L. starkeyi*) vary the preferred mechanism according to the temperature range considered [115].

The early studies on the ability of plants to acclimate to higher temperatures, conducted on plants adapted to high temperature growth [116,117], have shown decreasing level of unsaturated FA (e.g., 16:3) and increasing the level of saturated FA in their membranes at higher growth temperatures. Similar trends were later found for plants from temperate environments, which suggested that alterations in membrane lipids generally contribute to the ability of plants to acclimate to different temperatures [118,119]. The ability to adjust the degree of FA unsaturation and enable adaptation to high temperature is especially relevant in the membrane of thylakoids to ensure photosynthetic thermostability [120]. It is worthy of note that photosystem II (PSII) is embedded in the thylakoid membrane and that lipids play special roles in the assembly and function of PSII complex [121].

In fish, membrane FA composition has been related to membrane viscosity and it has been shown that red blood cells as well as neurons of adult carp can continuously adjust the fluidity of their external membranes to changing temperatures [122]. These authors, using mixtures of synthetic 18:1/22:6 phoshatidylethanolamines and 16:0/18:1 phosphatidylcholines demonstrated that these molecular species have the ability to increase membrane fluidity during adaptation to reduced temperatures. A comparable shift in FA membrane composition has also been observed in fish embryos exposed to pollutant 2,4-dinitrophenol related to unexposed embryos. Embryos exposed to this pollutant showed low PUFA level and increase in higher amounts of saturated FA, which increased the viscosity of the cellular membrane [123].

Other response to temperature changes that does not imply net lipid synthesis involves swapping the positions (remodelling) of attached FA in the glycerol backbone of phospholipids, which may alter the T_c_ by 8–9 °C [124,125]. Considering that the first and second *cis*-double bonds cause a massive change in the transition temperature (T_c_) of molecular species and additional double bonds have little or negligible effect in T_c_, accumulation of PUFA at low temperatures must be related to other advantages (see below) [119]. In homeotherms, for example mammals, the observed large variation in the acyl chain profiles of the membranes of several cell types and tissues, hints at the ability of these profiles to endow cellular membranes with specific properties. PUFA have a strong impact on other physical properties of membranes besides fluidity that encompass membrane permeability, membrane elasticity and curvature strain [126,127,128] with implications for membrane fusion and vesicle formation [129,130], lateral lipid segregation [131] and flip-flop mechanisms [132]. Due to the significance of lipid interactions for the formation of membrane domains, PUFAs modulate structure, organization and function of membrane rafts, interfere with acylated proteins in rafts and how lipids and proteins interact [133,134]. Additionally, EPA and DHA have been reported to modify membrane raft size, stability and distribution by decreasing the proportion of MUFA [135,136,137] and also affect membrane associated proteins such as signalling proteins and immunogenic receptors (see [138]).

From the biophysical perspective, the inclusion of PUFAs in phospholipid membranes also decreases its thickness and produces small defects in the geometrical arrangement of lipids [139]. These defects of different depth favour the binding and insertion of certain proteins with amphipathic α-helical conformation, depending on the bulkiness of their amino acid side chains [140,141]. PUFA are also important to maintain the spontaneous curvature and bending stiffness of membranes. By decreasing membrane bending rigidity [142,143], DHA in phospholipids was shown to promote rapid endocytosis [141] and arachidonic acid (20:4ω6) in membrane phospholipid of hepatocytes and enterocytes was shown to facilitate transport of triglycerides into the lumen of the endoplasmic reticulum [144].

In blood cells, the flexibility of membranes is increased in animals with a diet rich in fish oils (i.e., with abundant long-chain ω3 PUFAs such as EPA and DHA), with important consequences in microcirculation [145]. The decrease in the proportion of long-chain PUFA in cell membranes has been observed at preclinical stages of diabetes; it is suspected to reduce erythrocytes deformability and subsequent oxygen supply to tissues, thus promoting microvascular complications of diabetes and tissue hypoxia [146]. A decrease in erythrocyte long chain PUFA in phosphatidyl-ethanolamines has been observed in humans with diabetes and diabetic retinopathy, which seems to be compensated by the increase of the amounts of phosphatidyl-choline species in erythrocytes of diabetic patients without diabetic retinopathy [147]. It has been proposed that the increased release of free FA from adipose tissue into the circulation in type 2 diabetes and its prediabetic phase, gestational *diabetes mellitus* and obesity, elevates the plasma concentration of saturated FA (see [148]). This in turn, could create a shift from unsaturation into saturation of biological membranes, thus affecting their flexibility and functionality.

## 3. Energy Suppliers and Storage Material

### 3.1. In Prokaryotes

FA are used for the production of storage neutral lipids such as polyhydroxy alkanoates (PHAs), triacylglycerols (TAGs) and wax esters. Several prokaryotic species are able to accumulate large amounts of hydrophobic compounds as inclusion bodies in the cytoplasm, with only bacteria belonging to Actinomycetes being able to accumulate large amounts of TAGs as reservoirs for energy production and carbon [149,150]. The role of TAGs in cellular membrane fluidity regulation, by providing a storage place for unusual FA away from the membrane phospholipids or a sink for reducing equivalents, has also been discussed [150].

**Figure 1:**
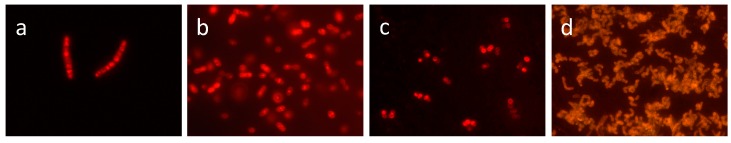
Accumulation of storage lipids in prokaryotes shown in Nile Red stained cells: PHA production in *B. megaterium* (**a**), *C. necator* (**b**) and *S. aureus* (**c**); TAG production in *R. erythropolis* (**d**); (de Carvalho and Caramujo, unpublished data).

Neutral lipid production, including PHAs, TAGs and wax esters, occurs in response to stress or imbalanced growth in prokaryotes, caused by e.g., depletion of nitrogen source or oxygen, in the presence of abundant carbon source (Figure 1). Accumulation of PHA was first reported in 1926 in *Bacillus megaterium*, which produced poly(3-hydroxybutyrate (PHB) [151]. However, the production of PHAs only regained interest in 1970s following the petroleum world crisis since the C4 homopolymer polyhydroxybutyrate may be used as a biodegradable thermoplastic. Besides, the physical properties of PHB biopolymers may be modified by the addition of copolymers such as 4-hydroxybutyrate and 3-hydroxyvalerate to reach properties comparable to polypropylene [152,153]. Of the hundreds of strains reported to produce PHAs, *Cupriavidus necator* (formally known as *Alcaligenes eutrophus* and *Ralstonia eutropha*) is the most studied bacterium [152,153]. PHAs act as carbon and energy reservoir, being composed of 3-hydroxy FA monomers that accumulate in intracellular granules coated with a layer of phospholipids and proteins [99,102], although the presence of phospholipids in PHA granules was recently questioned [154]. During PHA accumulation, *C. necator* cells are able to modulate the FA composition of the cellular membrane to promote the stretching of the membrane to accommodate the PHAs granules [155]. Accumulation of PHB is apparently common in bacteria growing in cold environments such as Antarctica as PHB may protect the cells from low temperatures and freezing [152,156]. Besides, 3-hydroxybutyrate not only protects the cells from thermal-mediated denaturation but also against oxidative damage induced by Cu^2+^ and H_2_O_2_ [157]. In fact, PHA accumulation has been reported in archaea and bacteria isolated from high salinity or extreme pH or temperature conditions, suggesting that PHA production at industrial scale may be achieved in a near future [156].

Wax ester synthase/acetyl-CoA:diacylglycerol acyltransferases (WS/DGAT), not related to known equivalent enzymes in yeast, plants and animals, have been identified in *Acinetobacter calcoaceticus* ADP1 and in several *Mycobacterium* species which are able to accumulate TAGs and wax esters [109,110]. Using fluorescent dyes, Wältermann et al. showed that prokaryotic neutral lipid accumulation starts at the cytoplasm membrane and only at a later stage do free cytoplasmic lipid-droplets appear [158]. The WS/DGAT was also shown to be located at the cytoplasm membrane in the same study. Some strains such as *Psychrobacter cryohalolentis* K5 and *Acinetobacter baylyi* have only a single copy of the WS/DGAT gene, whilst *Marinobacter aquaeolei* VT8 and *R. jostii* RHA1 contain multiple homologs for the WS/DGAT gene [159].

Wax esters, formed by a FA and a fatty alcohol linked by an ester bond, are produced by several bacterial species when grown on substrates such as hydrocarbons, alkanols and fatty acids under nitrogen-limiting conditions [113,114,115]. Their main function is as storage compound for both energy and carbon, yet wax esters may also act as a deposit for toxic or unused FA or as storage of evaporation-resistant lipids in case of desiccation [160].

TAG production in prokaryotes has been suggested to play several roles. In *Mycobacterium* cells, TAG and wax esters may be used for energy storage during long-term dormancy of *M. tuberculosis* [161,162]. *M. tuberculosis* cells exposed to the hypoxic environment of granulomas, in the human lung, accumulate TAGs by incorporation of FA from the host and enter a dormant state [163]. This bacterium is able to remain in a non-replicating drug-resistant dormancy state for decades until a frail immune system of the host allows its reactivation and the development of tuberculosis.

In *Rhodococcus* cells, TAGs are the main storage compounds and may contribute to the remarkable ability of these cells to grow and thrive on numerous carbon sources and environmental conditions [164,165,166,167]. Biosynthesis of TAG in *Rhodococcus* involves the conversion of diverse carbon sources to key metabolic intermediates including pyruvate, acetyl-CoA and glycerol-3-phospahe [167]. However, the cells may use different metabolic pathways for TAG production, depending on the carbon source used: cells grown on gluconate or glucose produce FA necessary for TAG via acetyl-CoA whilst cells consuming hexadecane as carbon source use the FA derived from the mono-terminal oxidation of the alkane [167,168]. One of the best studied *Rhodococcus* strains for neutral lipids accumulation is *R. opacus* PD630 since it accumulates TAGs but not PHAs. This strain can accumulate up to 76 or 87% of cellular dry weight as free fatty acids or acylglycerols when the cells grow on gluconate or olive oil as carbon source, respectively, under nitrogen limiting conditions [169]. Other *Rhodococcus* strains able to produce lipids that could be used for biodiesel blends include *R. opacus* B4 [170] and *R. opacus* PWD4 and *R. erythropolis* DCL14 [165].

### 3.2. In Eukaryotes

The oxidation of FA or beta (β)-oxidation, begins in the cytoplasm, where FA are converted into fatty acyl CoA molecules [41]. Fatty acyl CoA combines with carnitine to create a fatty acyl carnitine molecule and is transported across the mitochondrial membrane. Once inside the mitochondrial matrix, the fatty acyl carnitine molecule is converted back into fatty acyl CoA, which is then broken down completely by a cycle of reactions that cleaves two carbons at a time from its carboxyl end to generate one molecule of acetyl CoA for each turn of the cycle. During this process, one molecule of NADH and one molecule of FADH_2_ are produced. The newly formed acetyl CoA enters the citric acid cycle where the acetyl group is oxidized to CO_2_ and several molecules of the electron carrier NADH are generated in the same way as acetyl CoA derived from pyruvate.

When oxidation of FA is not necessary to produce energy, FA may be stored in the cells. Alternatively, the FA may originate from *de novo* FA synthesis, in which carbon must have been diverted from energy production to FAs biosynthesis. Once in the active pool, FAs can be esterified with glycerol or sterol backbones, generating triacylglycerols and sterol esters, respectively. These triacylglycerols and sterol esters are then surrounded by a phospholipid monolayer and specific proteins thus forming lipid droplets, which are ubiquitously present in eukaryotic and prokaryotic cells (see [171,172]. In a world of fluctuating energy supplies, the ability to store energy may provide a competitive evolutionary advantage to organisms. Lipid droplets are also a repository building material for phospholipids and sterols necessary to produce biological membranes and whose function is controlled in part by an evolutionarily-conserved protein network [171]. As amphipathic molecules, FA may compromise membrane integrity when abundant in the cell, while incorporated into triacylglycerol become considerably inert, stable and harmless (see [173,174]).

Intracellular structures that assimilate and traffic lipophilic cargo can be found in virtually every cell. Red/orange/yellow carotenoid pigments soluble in lipids, such as triacylglycerols or phospholipids, regardless of their polarity [175] are known to be stored in carotenoid droplets [176,177]. Carotenoids are synthesized by photosynthetic algae and plants, fungi and bacteria while other organisms must obtain the necessary carotenoids either directly from the diet, or through metabolic modification of the dietary carotenoid precursors. In plants and algae, carotenoids act as accessory light-harvesting pigments, participating in the collection of light energy and its transfer to chlorophyll for photosynthesis; and as photo-protectors of chlorophyll dissipating the excessive energy used in photosynthesis and inhibiting the formation of ROS [178,179]. The antioxidant properties of carotenoids are further extended to consumers of algae and plants, where they provide pigmentation, photo- and antioxidant protection (see [46]). Oxygenated derivatives of hydrocarbon carotenoids, or xanthophylls such as astaxanthin, are commonly esterified with FA. These FA are thus protected from peroxidation by the carotenoids, which are strong scavengers of free radicals [180]. Recent studies indicate that the accumulation of astaxanthin in zooplankton is strongly related to lipid metabolism, thus protecting lipids from peroxidation and enabling lipid use during periods of zooplankton rapid growth and reproduction [181,182].

Vitamins A, D, E and K are lipid-soluble vitamins that are stored within lipid domains. Vitamins E and K, synthesized by photosynthetic organisms and vitamin D which is synthesized along the sterol pathway by UVB exposure, are stored generally unmodified in lipid droplets [172]. Fatty acids are however involved in the transport and storage of vitamin A. β-carotene is the main source of vitamin A, or retinol. Retinol is formed by the enzymatic splitting of β-carotene into two molecules of retinal, an aldehyde that then undergoes reduction to retinol [183]. Retinol is then esterified to a fatty acid (e.g., 16:0) and may be stored as retinyl esters. Retinyl esters serve as the storage form of vitamin A, which in mammals is predominantly stored in the liver.

### 3.3. Sequestration of Toxicants in Lipid Droplets

Lipids also play a major role in the accumulation of lipophilic xenobiotic compounds such as PCBs, DDT and mercury (Hg) that have no physiological value for organisms [184]. FA may form FA conjugates of xenobiotics that accumulate in various organs and are involved in target organ toxicity [185]. Contaminants can be accumulated by organisms along the food web, especially when they are hydrophobic and are not readily biotransformed [186,187]. In humans, combustion-derived polynuclear aromatic hydrocarbons (PAHs) concentrate in respiratory cell lipid droplets and it has been hypothesized that these lipid droplets sequester toxicants in order to protect cells [188,189]. In cells, lipid bodies or lipid droplets containing neutral lipids such as TAGs and sterol esters have several important roles including membrane trafficking, recycling of phospholipids, intracellular protein metabolism, cell signalling and response to starvation [158,171,190]. One of the functions of lipid droplets is to accumulate lipids and proteins that may be considered toxic during metabolic stress and that may induce lipotoxicity and subsequent apoptosis for example, in yeast and mammalian cells [191].

As early as 1966, a relation between increased cellular lipid content and increase in resistance to various penicillins was reported in *B. subtilis*, *S. aureus* and *S. faecalis* [192]. The endolichenic fungus *Phaeosphaeria* sp., which produce phototoxic perylenequinones, trap these molecules inside lipid droplets since perylenequinones produce high quantum yields of reactive oxygen species (ROS) following exposure to light [193]. Inside TAG containing lipid droplets, fewer singlet oxygen molecules were generated by light irradiation of the perylenequinones. The same effect was observed for hypocrellin A, a phototoxic perylenequinone derivative and farnesol: trapped inside lipid droplets of *Candida albicans* and *Saccharomyces cerevisiae* these compounds lose the ability to induce the formation of ROS [193].

*R. opacus* PD630 is able to grow in phenyldecane, which belongs to the family of phenylalkane compounds that are common in crude oil and detergents and also to accumulate lipids derived from the catabolism of phenyldecane [194]. PD630 cells grown on this compound presented TAGs with two different compositions: one containing only odd- and even-numbered aliphatic FA; and another in which one FA is replaced by a phenyldecanoic acid resulting from oxidation of the substrate. TAG biosynthesis was investigated in *Nocardia globerula* 432, using the recalcitrant tetramethyl branched hydrocarbon pristine, under nitrogen depletion conditions which normally predominate in the environment [195]. The major compound produced was 4,8,12-trimethyl tridecanoic acid and this was the only branched FA in acylglycerols of strain 432, with mainly C16:0 and C18:0 being incorporated in TAGs. The cells were thus able to degrade a recalcitrant compound and to produce cellular lipids from its oxidation under growth limiting conditions.

## 4. The Roles of PUFA

ω3 PUFA are especially relevant for the normal growth and function of the human brain and retina, with new-borns with ω3 PUFA deficiency showing light sensitivity of retinal rod photoreceptors significantly reduced [196]. DHA is the predominant FA found at the sn-2 position in the phospholipids of neuronal and synaptic membranes [197]. In vertebrates, DHA acts as a neurotrophic factor [90], modulates synaptic activity [198] and is involved in anti-inflammatory signalling ([199,200,201], see below). A diet rich in ω3 has since long been connected with maintaining cardiac and vascular health and preventing atherosclerosis [202,203,204]. Although some gaps in knowledge remain regarding the precise mode of action explaining the cardioprotective effects of EPA and DHA, several mechanisms have been proposed: preventing arrhythmias [205,206], lowering plasma triacylglycerol [207], preventing chronic intermittent hypoxia-induced atherosclerosis [208], decreasing arterial cholesterol delivery and preventing the formation of atherosclerotic plaques in arteries [209], regulating vascular endothelial cell function and improving vascular relaxation [210], decreasing platelet aggregation [211] and arterial inflammatory responses [212].

As referred in previous sections, the molecular balance of PUFA in cell membranes is influenced by environmental, metabolic and nutritional conditions (see also [213]). In turn, the PUFA composition of membranes in animals has important consequences for tissue homeostasis and physiological functions and condition the balance of PUFA derived signalling molecules.

Lipids that act as extracellular and intracellular messengers to control cell metabolism in normal physiology and disease include a wide range of lipid classes, such as lysophospholipid, sphingolipid, phosphatidic acid, inositol phosphate, diacylglycerol, *N*-acylethanolamine, fatty acids, oxylipins. Although FA are molecular moieties of other lipid classes, here we will focus on the signalling roles of physiologically active fatty acids and their oxidized products, oxylipins.

In the early 1960’s, the identification of prostaglandins as signalling molecules derived from 20:4ω6 FA [214,215] led to the discovery of a class of oxygenated metabolites of C20 PUFA named eicosanoids [216]. The various groups of eicosanoids encompass the prostaglandins, which originally were shown to be synthesized in the prostate gland [217], the thromboxanes that originated from platelets (thrombocytes) and the leukotrienes from leukocytes, hence the derivation of their names [218]. Eicosanoids have a large range of biological functions, including promoting and enhancing inflammatory responses, regulating pregnancy and child-birth, controlling blood pressure and the secretion of stomach mucus and acid, contracting or relaxing smooth muscle.

Prostaglandins and leukotrienes have been largely appreciated for their activities promoting and enhancing inflammatory responses. Through the sequential actions of prostaglandin G/H synthase (or cyclooxygenase, COX-1 and COX-2) and respective synthases, various cell types (e.g., leukocytes) rapidly synthesize these lipid mediators from 20:4ω6 released from the membrane by phospholipases (PLA_2_) within seconds to minutes of an acute stimulus [218]. Products of COX-2 in particular may also contribute to resolution of inflammation in certain settings [219]. The resolution of inflammation, long considered to be a passive process, is now known to be highly regulated, following a complex program involving pro-resolving mediators that promote the return to tissue homeostasis [220]. As leukocytes (neutrophils, eosinophils and basophils) recede from sites of acute inflammation and the levels of proinflammatory cytokines and chemokines decrease, a switch of arachidonic acid–derived prostaglandin and leukotriene synthesis to pro-resolving mediators, such as lipoxins, occurs [221]. Lipoxins, together with the (neuro)protectins, resolvins and maresins, which are generated from ω-3 PUFA precursors can orchestrate the timely resolution of inflammation [220,221,222]. (Neuro)protectins, resolvins and maresins are oxygenated metabolites (i.e., oxylipins) derived mainly from eicosapentaenoic acid (20:5ω3 or EPA), docosapentaenoic acid (22:5ω3) and especially docosahexaenoic acid (22:6ω3 or DHA).

The biological significance of eicosanoids has been largely focused on the human or mammal pathophysiological context. Nevertheless, eicosanoids and other oxylipins (i.e., oxygenated metabolites of FA), have been recorded in unicellular organisms and invertebrates [223,224], plants and algae [225,226,227] and are recognised as important regulators at the ecological level of organisation (see [228,229]). Depending on the timing, location and level of release, the FA-derived signal may act as a toxin, deterrent, pheromone, or growth resource.

FA and their derivatives function as allelopathic agents in a number of algal systems, possibly through the modification of chloroplast membrane characteristics, inhibiting photosynthesis and disrupting the permeability and integrity of plasma membrane [230,231]. Numerous studies have implicated FA as toxigenic agents in food web interactions, with various algal groups having the ability to release free FA that damage grazer tissues, are embryotoxic and reduce overall reproductive success of grazers [232,233,234,235]. As noted by Watson et al., much of the semiochemical activity attributed to oxylipins in both terrestrial and aquatic food webs is accounted by polyunsaturated aldehydes (PUA) and volatile unsaturated hydrocarbons derived from PUFA [228]. PUA are produced by plants, macroalgae and microalgae. Formation of PUA occurs following wound-activation (e.g., by grazers) and is under the control of lipolytic enzymatic activity [236,237,238]. The bioactivity of PUA is, in general, manifested as reduced fecundity and hatching success of herbivores, not as reduced adult survivorship. PUA impact is exerted on the reproductive and developmental processes, encompassing oocyte maturation, sperm motility, fertilization, embryogenesis and larval competence [239,240,241,242]. This bioactivity led to the suggestion that the production of PUA could facilitate bottom-up control of grazer populations by limiting recruitment through reproductive interference [225,243].

## 5. Biomarkers of Organisms

The great structural diversity and substantial taxonomic specificity of FA in bacteria and eukaryotic organisms confers biomarker property to some individual FA and FA ratios. This property has been explored, increasingly with the help of multivariate statistics, to identify organisms [244], to infer the species composition of assemblages [245], to trace carbon cycling and the transfer of materials in food webs [246,247] and to disclose certain environmental processes.

### 5.1. Identification of Prokaryotes by FA Fingerprint

Microbes may be identified using DNA sequencing, biochemical tests and FA profiling. All systems have strong points and limitations [248,249,250]. Tang et al. compared phenotypic and genotypic techniques to identify 72 unusual aerobic pathogen Gram-negative bacteria isolated from samples collected at the Mayo Clinic [250]. They were able to identify 56, 63 and 70 isolates at genus level using fatty acid profiling, biochemical reactions and 16S rRNA sequencing, respectively and 44, 55 and 58 isolates out of 65 at species level.

The first report that cellular FA analysed by gas chromatography could be used to identify bacteria was made in 1963 by Abel et al. [251]. Bacteria contain ca. 5–10% of dry weight as lipids and usually FA with chains containing 9–20 carbon atoms. Since FA may be saturated, unsaturated, linear, branched, containing hydroxyl or methyl groups or cyclopropane groups, each bacterial species has a specific profile that acts as a fingerprint. It has been noted that the identification obtained by FA profile sometimes is not in agreement with biochemical criteria in strains of the genera *Acinetobacter*, *Moraxella*, *Alcaligenes* and *Pseudomonas* [250,252], probably due to differences in culture age at the time of harvest, insufficient characterization of FA libraries entries, and/or inability to differentiate chemotaxonomically closely related species [252]. However, when the growth conditions are standardized in terms of culture medium composition, incubation temperature and physiological age of the cell population, cellular FA profiles are reproducible and accuracy of identification increases [248] enabling the use of automated systems such as the Microbial Identification System (MIS; MIDI, Inc., Newark, DE, USA) [253]. When this system was compared with a self-generated library made with strains from the National Collection of Plant Pathogenic Bacteria (UK) for the identification of 773 strains, both provided good accuracy although a slightly higher level was observed with the latter library since it only contained plant pathogenic species and the diagnosis time could be reduced from an average 2–3 weeks to 3–4 days [254]. The system also exhibited an excellent correlation with pulse-field gel electrophoresis for the identification of methicillin-resistant *Staphylococcus aureus* during an infection outbreak [255]. Additionally, both systems allowed the identification of two isolates, previously thought to be epidemiologically associated, as different from the clustered strains. In fact, the MIS could be efficiently used to assess the epidemiology for cross-infection or outbreaks of infections caused by coagulase-negative staphylococci, with 200 isolates from 5 hospital culture collections being fully characterised in 5 days by one person [256].

The application of FA profile for identification of bacteria may be particularly useful when members of different species present high rDNA similarity, such as in the case of the genus *Bacillus*. Species of *B. anthracis*, *B. cereus*, *B. mycoides* and *B. thuringiensis* constitute an interesting taxonomic problem since they share a very high resemblance of 16S rRNA and DNA sequence homology [257,258] and minor differences in 16S to 23S ribosomal intergenic spacer sequences [259]. However, FA analysis enabled the correct taxonomic identification of *B. mycoides* strains in a large set of isolates, with the results being supported by 16S rDNA analysis and hybridization with an oligonucleotide probe [260]. Using 1071 FA profiles covering a genus-wide spectrum of 477 strains and 82 species of *Bacillus*, Slabbinck et al. applied an artificial neural network approach which could further improve the identification of *Bacillus* species [261].

Curiously, a Cauliform bacterium, which has prosthecae as cellular appendages, from an abyssal hydrothermal vent showed no detectable phospholipids or sulpholipids but contained 1,2-di-*O*-acyl-3-*O*- α-d-glucopyranosylglycerol, 1,2-di-*O*-acyl-3-*O*-α-d-glucopyranuronosylglycerol and the novel lipid 1,2-di-*O*-acyl-3-[*O*-α-d-glucopyranuronosyl]glycerol-6′-*N*-glycine [262]. These glucoronosyl lipids are presumably replacing phospholipids in the cell. The strain, proposed to represent a novel species of a new genus, *Glycocaulis abyssi*, contained as main FA C18:1ω7 *cis*, C18:0, an unknown FA and C12:0 3-OH. Polar lipids may also differentiate Archaea organisms at the genus and sometimes at species levels [263,264,265]. The Subcommittees on the taxonomy of *Halobacteriaceae* and *Halomonadaceae* even suggest that fatty acid analysis “should be added as required rather than recommended” for describing new taxa in *Halomonadaceae* [264].

In 1979, White et al. showed that the analysis of phospholipids analysis and of polar lipid fatty acids (PLFAs) could provide valuable information to estimate the microbial biomass and the microbial community structure in marine and estuarine sediments [266]. This method could overcome difficulties associated with conventional enumeration and isolation of microorganisms in the laboratory. Since phospholipids from cellular membranes are rapidly degraded to neutral lipids upon death of the organism, PLFA analysis provide valuable information of the living biomass of a given niche, without the necessity to cultivate the organisms [266,267,268]. DNA is not easily extracted from e.g., soil samples, has a longer persistence after cell-death thus providing information also of non-viable biomass and knowledge about target sequences for PCR amplification is needed beforehand [269]. Furthermore, when comparing PLFA results with those obtained with adenosine tri-phosphate, flow cytometry and quantitative real-time PCR, Zhang et al. showed that PLFA results correlated with the other three methods while presenting lower variation than that of quantitative real-time PCR [270]. Besides being a rapid and cheap technique, PLFA could differentiate shifts in the microbial communities that were not detected by community level physiological profiling (in 14 of 32 studies) or PCR-based molecular methods (in 5 or 25 studies) [271].

Frostegård et al. showed in 1993 that PLFA composition of soils could be used to study the shifts in the structure of soil microbial communities [268], while White and Ringelberg published detailed methodology to use PLFA analysis to answer fundamental questions in soil science [272].

### 5.2. FA Markers in Eukaryotes and the Use of FA Profiles to Assess Species Composition of Assemblages

In eukaryotes, FA cannot be used as taxonomic indicators at the species level, which contrasts with their use in prokaryotes. However, the presence and combinations of certain FA are often characteristic of particular algal classes and of groups of other organisms. Since the 1960’s, laboratory studies have examined the FA composition of marine microalgae (e.g., [273]) and it was shown that each class of microalgae is characterized by a specific fatty acid profile: 18:5ω3 are present in Dinophyceae and Haptophyta I; 16:2ω4, 16:3ω4, 16:4ω1 and 20:5ω3 in Bacillariophyceae; 16: 3ω3, 16:4ω3 and 18:3ω3 in Chlorophyta; a high proportion of 18:1ω9 and 18:2ω6 and lack of long chain PUFA in Cyanobacteria [245,274,275,276,277,278,279,280,281]. The algal lipid biochemistry is nevertheless widely influenced by the growth and environmental conditions and the proportion of FA is often altered under salinity, light, temperature and nutrient fluctuating conditions (see [282,283]).

Specific fatty acids and fatty acid ratios have been applied to evaluated the taxa composition of plankton communities, often resorting to multivariate analysis methods [245,284,285]. The use of FA to derive the taxa composition of the lower levels of the aquatic communities still presents relevant challenges when heterotrophic protists are included [284,286]. The FA composition of heterotrophic protists (e.g., ciliates and flagellates) is highly variable and is thought to be mostly influenced by their habitat (marine vs. freshwater) and their diet (bacterivorous vs. algivorous) (see [287]). In general, freshwater heterotrophic protists contain high amounts of ω6 FA while marine ciliates contain high levels of ω3 FA. The presence of high levels of ω3 FA in both marine and freshwater algivorous heterotrophic protists has been associated with accumulation of dietary PUFAs.

The terrestrial contribution to aquatic ecosystems has also been assessed through the use of 18:2ω6, 18:3ω3, 22:0, 24:0 FA as markers of terrestrial plant material [288,289]. The use of FA markers is especially interesting to assess the relative contributions of different food sources to the diet of consumers and its application ranges from the assessment of the impact of algal FA on herbivorous zooplankton lipid content [275] to the inspection of niche separation in pinnipeds (seals and walruses; [290]).

### 5.3. Using FA to Follow Energy Fluxes in Food Webs

The application of PLFA analysis has been especially useful to estimate the type and numbers of organisms in a community and to monitor and understand the transfer of energy through food webs. To disclose the trophic relationships in food webs it is important to consider that in general, the FA pattern of neutral lipids in the consumer is more likely to reflect that of the dietary items, while the FA composition of phospholipids (PL) is probably under metabolic and abiotic control and thus is weakly influenced by dietary FA [246,275,291]. Nevertheless, the consumer metabolism influences the uptake, incorporation, modification and deposition of dietary FA in the organism’s reserves and neutral FA composition of the predator will never exactly match that of its prey (see [292,293]). Additional analytical challenges arise when using FA to disclose trophic relationships in higher organisms (e.g., from fish to seals to bears) along the food web as the FA composition of the predator neutral lipids must be analysed to disclose its diet and the total FA composition must be assessed when the same organism becomes a prey for the next trophic level. Nevertheless, top-predators are integrators of lower level energy fluxes in food webs and the analysis of their FAs may disclose trophic pathways and improve the knowledge on mass transfer through predator-prey interactions (e.g., [294]). It is noteworthy that FA markers can be further used to disclose cross-ecosystem fluxes [295].

As a point of departure, several FA may be used as biomarkers of certain taxa although researchers should be aware that most FA are not exclusive of an organism (Table 3). For example, cyclopropyl FA such as cyclo17:0 and cyclo19:0 are associated to anaerobic bacteria but can also be found in older cells of Gram-negative bacteria. These cells convert 16:1ω7c and 18:1ω7c to, respectively, cyclo17:0 and cyclo19:0 as they move from exponential to stationary growth phase [79,172]. The ratios between the different FA present in the organisms are therefore more reliable for their identification than the use of individual markers [179,199,224]. This alternative approach to relate FA profiles with taxa involves the use of multivariate statistical methods such as principal component and discriminant analysis and partial least squares analysis [281,292,296,297,298]. Databases using FA profiles of e.g., aerobic and anaerobic bacteria and algal classes can be made and multivariate tools can be applied to determine the presence of groups of organisms in sampled assemblages from their “bulk” FA profiles [299,300].

**Figure 2:**
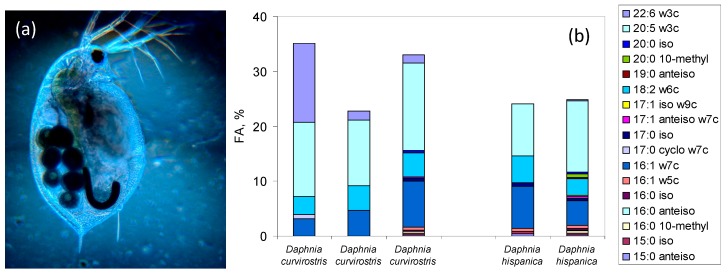
(**a**) In temporary Mediterranean water ponds, *Daphnia* sp. feeds on bacteria, fungus and algae—algal material visible as green mass inside the gut; (**b**) FA of auto and heterotrophic origin are incorporated into the phospholipids of *Daphnia*, as shown by PLFA analysis (de Carvalho and Caramujo, unpublished data).

Papers by Dijkman & Kromkamp [245] and ourselves [298], have shown that a matrix factorization program, CHEMTAX, could be used to quantify phytoplankton composition and the auto- and heterotrophic biomass at the base of the food web of temporary Mediterranean ponds, using the relative proportion of specific FA derived from polar lipids (PLFA). The software, which was initially developed to calculate algal class abundances from chlorophyll and carotenoid measurements, was adapted to use FA data as the input ratio matrix and determined iteratively which ratios best fitted the data to determine group abundance from the PLFA composition of each temporary pond [298]. Further comparisons with the FA composition of zooplankton showed that zooplankton consumed and incorporated bacterial FA into their body tissues, including into their phospholipids, indicating that not only materials of autotrophic origin but also materials of heterotrophic origin contributed to the aquatic freshwater food web (Figure 2).

FA markers are important to assess relative contributions of biomass of different origin to different organisms in a community in different seasons. For instance, in a small forested stream, the FA content and profile of: (i) autochthonous food sources such as periphyton, green algae, red algae and bryophytes; (ii) allochthonous food resources resulting from benthic and transported organic matter; and (iii) macroinvertebrate consumers, can be used to evaluate the temporal contribution of food sources to provide essential FA to each group of macroinvertebrates [301]. Nonmetric multidimensional scaling analysis of the FA showed that the mayflies of the genera *Ephemerella* and *Hydropsyche* consumed mainly autochthonous food sources even during the shaded summer period, while isopods and oligochaetes consumed a mixed diet of terrestrial matter and algae. The pulse of ω3 FA from diatoms and other algae may be of paramount importance for macroinvertebrate health and reproduction suggesting that autochthonous food sources in forested streams may be more relevant than initially considered.

The importance of prokaryotes and in particular of bacteria, in aquatic food webs has been increasingly highlighted in lipid studies [98,302]. Prokariotes are key dietary items for omnivorous, sestonivorous and filtering benthic animals and they may be a significant source of PUFA in marine microbial niches including abyssal communities, sea ice and in marine animals [98]. Prokaryotes are particularly important in extreme environments, such as hydrothermal vents, since bacteria and archaea may adapt their cellular membranes and produce specialize lipids that allow their survival in these niches, becoming the major food source available for crustacean species [302].

Stable isotope analysis is a versatile tool that may be applied to study the flow of energy and matter among organisms and estimate their trophic level [303]. It may be used in parallel with FA biomarker analysis or, the ^13^C composition of individual FA may be analysed through compound-specific carbon isotope analysis to disclose details of compound transfer from one trophic level to the following. As an example, the efficiency and type of compound transfer at the plant–animal interface of aquatic food webs by e.g., copepods that are both consumers of primary production and a food source for higher trophic levels, may thus be assessed. In laboratory grazing experiments where the harpacticoid copepod *Microarthridion littorale* grazed for 9 days on ^13^C labelled diatoms and ^13^C labelled bacteria, it was possible to infer the magnitude and relevance of FA transfer between consumer and dietary items [304]. Copepods feeding on bacteria that did not produce DHA and where EPA was less than 5% of total FA, still presented labelled DHA in small quantities, which indicated a limited ability to elongate short length PUFA available in the bacteria. When compared to field specimens, copepods feeding on either bacteria or diatoms presented a lower proportion of C18 FA and increased proportion of both EPA and DHA. This suggested that copepods must have been able to elongate other FA to EPA and DHA, suggesting that harpacticoid copepods may elongate FA and exploit niches with poor quality food and contribute to trophic upgrading of biochemicals in food webs. 

## 6. Conclusions

Fatty acids are important “building” blocks in Nature. The number of fatty acyl molecules presently in the Lipid Maps Structure Database [320] surpasses 7200. As the number of e.g., bacteria and archaea isolated from extreme environments increases, better analytical tools are available and our understanding of lipid metabolism in both prokaryotes and eukaryotes widens, FA with novel structures and/or roles will likely be found. At the moment, their role in the regulation of the cellular membrane fluidity in all cell types and their participation in complex processes in eukaryotes such as proliferation, differentiation, secretion, migration, invasion and phagocytosis, highlights the importance of these molecules to the maintenance of life on Earth. Knowledge on the implications to human health of the type of dietary FA ingested and the understanding of the benefits of a ω3 PUFA-rich diet should help the general population to live a longer and healthier life. The examples of FA intervention in life processes discussed in this paper support the relevance and importance of further research in the fields of e.g., lipidomics and metabolomics for the full elucidation of the multiple roles of FA in the cellular/organismal/ecological realms.

## Figures and Tables

**Table 1:** Examples of lipid classes and representative molecules. PE—Phosphatidylethanolamine; PC—Phosphatidylcholine; TG—Triacylglycerol; WE—Wax ester.

Lipids	Example	Role	Specificities
Fatty acids	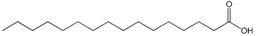 16:0	Building blocks for numerous lipids, regulation of membrane fluidity	-
Glycerophospholipids or phospholipids	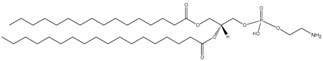 PE (16:0/18:0)	Main constituent of cellular membranes in prokaryotes and eukaryotes	Two FA linked to a glycerol molecule connected to a phosphate head group
Glycerophospholipids plasmalogens	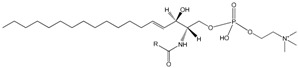 Sphingomyelin	Organization and stability of membranes; cellular signalling	Contain a vinyl-ether and an ester bond at the glycerol backbone
Glycerophospholipids sphingolipids	Sphingomyelin	Role in cell division, differentiation and cell death	Long-chain or sphingoid base linked to a FA via an amide bond
Glycerolipids	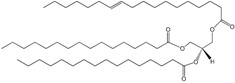 TG (16:0/16:0/18:1)	Storage compounds in prokaryotes	Mono-, di-, or tri-substituted glycerols
Ether-linked lipids such as glycerol dibiphytanyl glycerol tetraethers	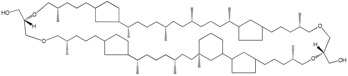 Crenarchaeol	Core cellular membrane lipids in many archaea	Isoprenoid moieties linked by ether bonds to glycerol
Wax esters	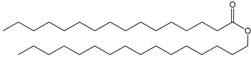 WE(16:0/16:0)	Energy storage and cell structure	Ester of FA and a fatty alcohol
Sterol lipids	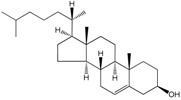 Cholesterol	Maintenance of membrane fluidity	Steroids with a hydroxyl group at the 3-position of the A-ring

**Table 2:** Modifications carried out by prokaryotes in the fatty acid composition of the phospholipids of the cellular membrane under varying growth conditions.

FA Modification	Effect	Microorganism	Reference
fatty acyl chain length	growth at different temperatures	*Micrococcus cryophilus*, *Shewanella oneidensis*, *Escherichia coli*	[49,50,51]
	growth in deep-sea	barophilic bacteria	[52]
	adaptation to the presence of organic compounds	*Rhodococcus erythropolis*	[53]
unsaturation	growth at different temperatures, pH, pressure, salinity, in the presence of organic solvents	archaea and bacteria	[43,54,55]
polyunsaturation	growth in deep-sea	*Alteromonas* sp., *Shewanella* sp.	[56,57,58]
	rapid adaptation to increased salinity and extreme conditions	*Rhodococcus erythropolis*	[59,60]
*cis-trans* isomerization	fast adaptation to environmental conditions when growth is inhibited	*Pseudomonas* and *Vibrio*	[61,62]
branching at *iso* or *anteiso* position	growth at different temperatures	*Listeria monocytogenes*	[63]
	persistence to high concentrations of antibiotics	*Staphylococcus aureus*	[64]
	growth temperature and presence of phenols	*Arthrobacter chlorophenolicus*	[65]
cyclopropanation	persistence and virulence of the cells	*Mycobacterium tuberculosis*	[66]
	osmotic tolerance	*Pseudomunas halosaccharolytic*	[67]
	growth at different temperature and pH	*Rhodococcus erythropolis, Salmonella typhimurium*	[60,68]
polyunsaturated fatty acids associated to phosphatidylglycerol	growth in the Marianas Trench at 11,000 m	Barophilic bacteria *DB21MT-2 and DB21MT-5*	[51]
composition of the alkyl and acyl chains in glycerol ether lipids	growth at different temperatures	*Desulfatibacillum aliphaticivorans*, *D. alkenivorans*, *Thermodesulfobacterium commune*	[69]

**Table 3:** Fatty acids used as taxonomic biomarkers (adapted from [298].

Fatty Acid	Category	Reference
*Mono-Unsaturated Fatty Acids (MUFA)*		
16:1ω7c	Bacteria Bacillariophyceae (diatoms) Cyanophyceae (cyanobacteria) Prymnesiophyceae	[245,274,292,305]
16:1ω5c	mycorrhizal fungi	[306]
16:1ω8c	Type I methanotrophs (gamma-proteobacteria)	[307,308]
17:1	Cyanobacteria	[309]
17:1ω6c (up to 60%)	*Desulfobulbus, Desulforhabdus, Desulforhopalus* (sulphate reducing bacteria)	[310]
18:1ω9c	Chlorophyceae (green algae) Cyanophyceae Dinophyceae Prymnesiophyceae Gram-positive bacteria	[245,274,292,305,311]
18:1ω7c	Bacillariophyceae (up to 10-fold more 18:1ω7c than 18:1ω9c) Cryptophyceae Cyanophyceae (less amount than 18:1ω9c) Prymnesiophyceae	[245,274,292]
18:1ω7t	Gram-negative bacteria	[311]
18:1ω8c	Type II methanotrophs (alpha-proteobacteria)	[307,308]
*Hydroxy substituted Fatty Acids (OH FA)*		
(e.g., 3-OH 10:0)	Gram-negative bacteria	[312]
*Cyclopropyl saturated Fatty Acids (cyFA)*		
(e.g., cyclo17:0, cyclo19:0)	Gram-negative bacteria, anaerobic bacteria	[313,314]
Cyclo17:0ω5,6	*Desulfosarcina/Desulfococcus* (sulphate reducing bacteria)	[310]
Iso*- and* anteiso*-branched Fatty Acids*		
(e.g., *iso*-15:0, *anteiso*-17:0) *iso*-17:1ω7c	Gram-positive bacteria Sulphate reducing bacteria *Desulfovibrio* sp. (sulphate reducing bacteria)	[313,314,315,316]
*Methyl-branched Fatty Acids (10-Me FA)*		
10-Me 18:0 10-Me 16:0 2-Me 17:0	Actinomycetales (Actinobacteria) *Desulfobacter* sp.- sulphate reducing bacteria Cyanobacteria	[309,315,316,317]
*Furan Fatty Acids*		
Fu18:2ω6, Fu17:2 ω5 and ω6	*Dehalococcoides* sp.	[318]
*Polyunsaturated Fatty Acids (PUFA)*		
16:2ω7	Bacillariophyceae	[245]
16:2ω6	Chlorophyta	[245]
16:2ω4	Bacillariophyceae Prasinophyceae	[245,279]
16:3ω4	Bacillariophyceae	[245,279]
16:3ω3	Chlorophyta	[245]
16:4ω3	Chlorophyceae Prasinophyceae	[245]
16:4ω1	Bacillariophyceae (diatoms)	[245,279]
18:2ω6	Chlorophyta Cyanophyceae (freshwater) Dinophyceae Prymnesiophyceae Fungi	[245,274,292,305,313,314]
18:3ω6	Cyanophyceae (freshwater) Saprophytic fungi	[274,314,319]
18:3ω3	Chlorophyceae Crypophyceae Cyanophyceae Dinophyceae Prasinophyceae Prymnesiophyceae	[245,274,292]
18:4ω3	Most groups (both marine and freshwater)	[245,274,292]
18:5ω3	Dynophyceae	[245]
20:4ω6	Bacillariophyceae Rhodophyceae	[245]
20:5ω3	Bacillariophyceae Cryptophyceae Dinophyceae Pavlovophyceae Rhodophyceae	[245,292]
22:5ω3	Bacillariophyceae Cryptophyceae Prasinophyceae	[245]
22:6ω3	Bacillariophyceae Cryptophyceae Dinophyceae Haptophyta (Prymnesiophyceae and Pavlovophyceae)	[245,274,292]
